# Recurrent hypertrophic pulmonary osteoarthropathy in an adult with bronchiectasis

**DOI:** 10.1002/rcr2.602

**Published:** 2020-06-23

**Authors:** Amelia Tekiteki, William R. Good, Benjamin Diggins, Graeme Anderson, Conroy A. Wong

**Affiliations:** ^1^ Department of Respiratory Medicine Middlemore Hospital, Counties Manukau DHB Auckland New Zealand; ^2^ Department of Medicine, Faculty of Medical and Health Sciences The University of Auckland Auckland New Zealand; ^3^ Department of Radiology Middlemore Hospital, Counties Manukau DHB Auckland New Zealand

**Keywords:** Bronchiectasis, clubbing, hypertrophic pulmonary osteoarthropathy

## Abstract

Hypertrophic pulmonary osteoarthropathy (HPOA) is a well‐documented complication of pulmonary malignancy and cystic fibrosis (CF). However, HPOA associated with exacerbations of non‐CF bronchiectasis has only been reported once previously in an adolescent. We describe a case of an adult patient with bronchiectasis and HPOA, whose joint symptoms flared during pulmonary exacerbations and improved with treatment of each exacerbation.

## Graphical abstract

We describe a case of an adult patient with bronchiectasis and hypertrophic pulmonary osteoarthropathy (HPOA), whose joint symptoms flared during pulmonary exacerbations and improved with treatment of each exacerbation.

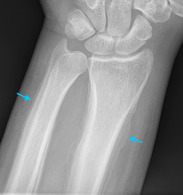

## Introduction

Hypertrophic pulmonary osteoarthropathy (HPOA) is characterized by clubbing, periostitis of tubular bones, and arthritis‐like symptoms and signs. The association between HPOA and pulmonary malignancies is well documented in the literature [1]. It is also a well‐recognized cause of arthropathy in cystic fibrosis (CF) [2], but its association with non‐CF bronchiectasis is extremely rare. We report a case of HPOA in a 30‐year‐old male with bronchiectasis, who had recurrent periostitis with each exacerbation of bronchiectasis. We believe that this is the first case of HPOA secondary to bronchiectasis to be described in an adult.

## Case Report

A 30‐year‐old Cook Island Māori male patient was admitted to hospital with a one‐week history of cough, purulent sputum, minor haemoptysis, and fever. He described bilateral, symmetrical polyarthralgia involving the elbows, wrists, knees, and ankles.

He was diagnosed with bronchiectasis at the age of 16 years with a presumed post‐infective aetiology following adenovirus infection in early childhood. A sweat test was performed at the age of 16 years, which was negative, and further genetic testing for CF, performed following this recent admission, detected no pathogenic cystic fibrosis transmembrane conductance regulator (CFTR) gene mutations. He also had a history of bilateral sinonasal polyposis but a diagnosis of primary ciliary dyskinesia was considered to be unlikely as he was not infertile.

In the two years preceding this admission, he had experienced three similar episodes of symmetrical polyarthralgia, each occurring during an infective exacerbation of bronchiectasis. *Proteus mirabilis* was repeatedly cultured in sputum samples over this period. Upon treatment of his respiratory infection with antibiotics, his joint symptoms subsided. He required morphine for his joint pain but was not treated with prednisone during these previous flare‐ups.

**Figure 1:**
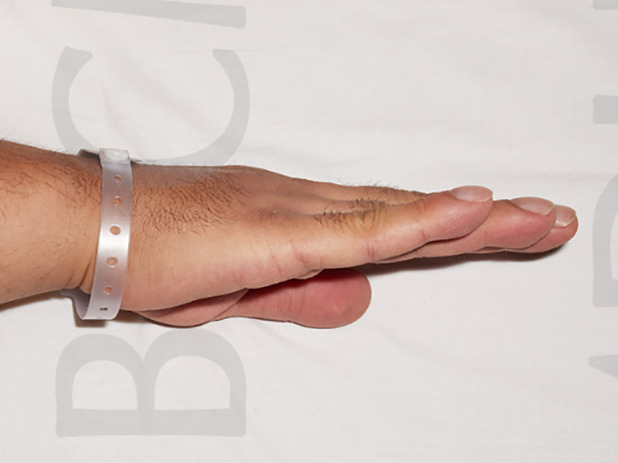
Digital clubbing.

Physical examination revealed bilateral, symmetrical swelling of the wrist, knees, and ankles. They were tender and warm to touch but had a full range of passive movement, and no overlying skin changes were present. Temperature was 38.8°C and respiratory rate was elevated at 32/min. Pulse oximetry revealed oxygen saturation of 93% on room air. Body mass index was 37 kg/m^2^. Grade 5 symmetrical digital clubbing was present (Fig. 1). Auscultation of the lung fields revealed bilateral coarse crackles.

Admission laboratory investigations revealed haemoglobin of 146 g/L, white blood cell count 9.0 × 10^9^/L, calcium 2.30 mmol/L, C‐reactive protein 147 mg/L, and alkaline phosphatase of 297 U/L. Serology for rheumatoid factor, extractable nuclear antigens (ENA), anti‐double stranded DNA, and anti‐cyclic citrullinated peptide was normal. Sputum cultures identified *P. mirabilis* but no mycobacteria. Intra‐articular aspiration of both knees revealed no evidence of joint infection or crystal arthropathy. Spirometry three months prior to admission showed forced expiratory volume in 1 sec (FEV_1_) of 1.75 L (43% predicted), forced vital capacity (FVC) 2.78 L (57%), and FEV_1_/FVC of 63%. Gas transfers demonstrated a diffusion capacity of the lungs for carbon monoxide (DLCO) of 55% and carbon monoxide transfer coefficient (KCO) of 126%.

**Figure 2:**
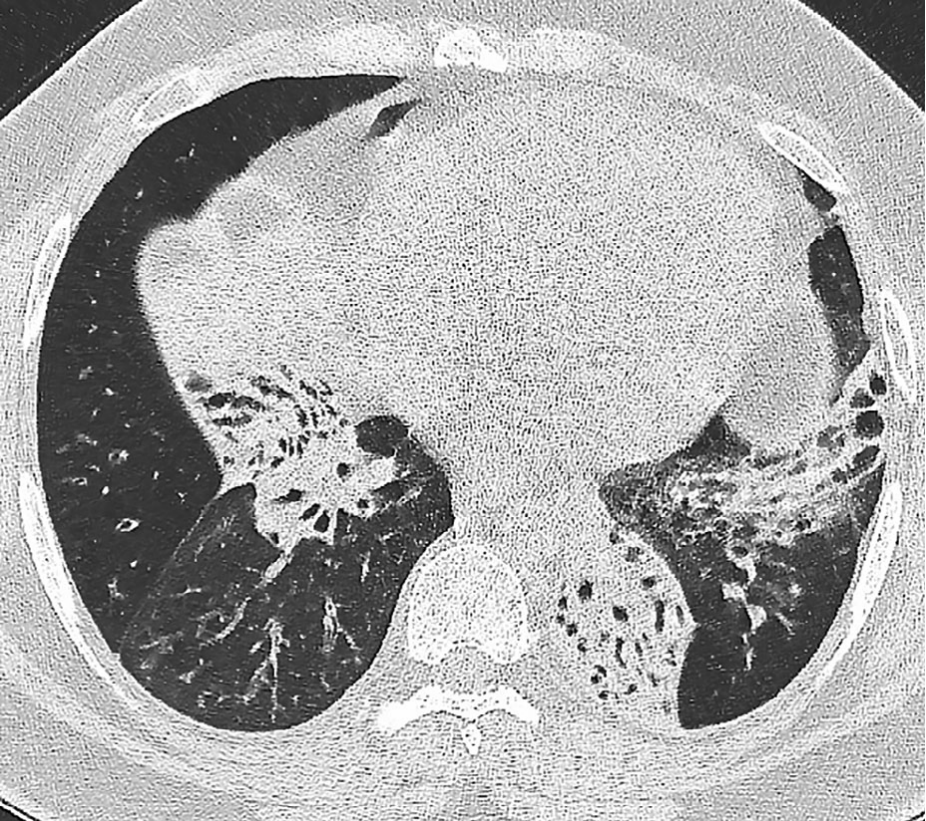
High‐resolution computed tomography (HRCT) demonstrating bilateral bronchiectasis and atelectasis.

**Figure 3:**
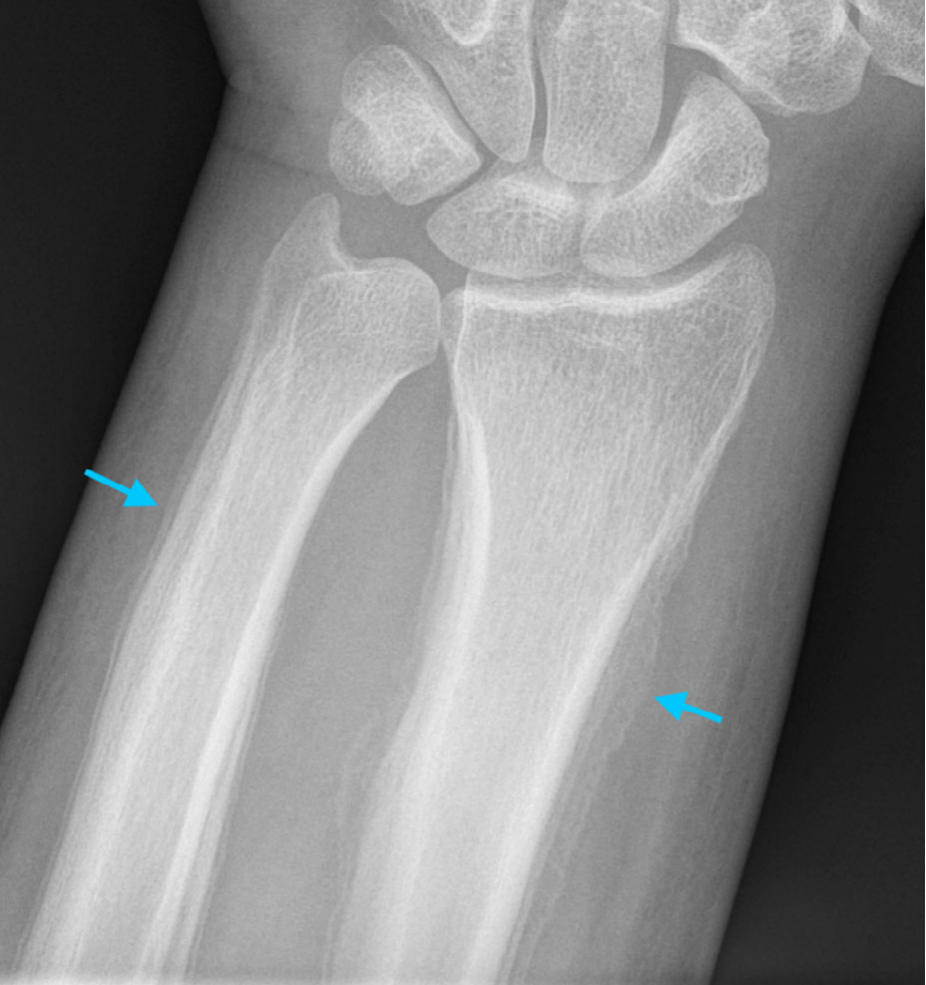
Left wrist radiograph demonstrating florid periosteal new bone formation (blue arrows): distal radius and ulna.

**Figure 4:**
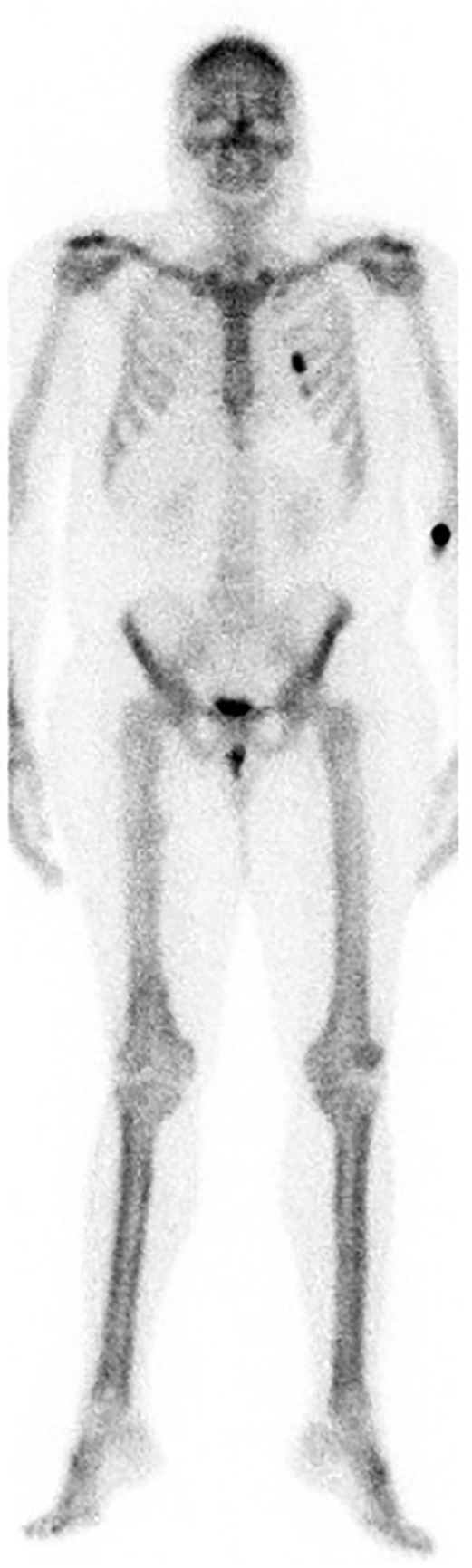
Technetium 99 bone scan showing smooth diffuse periosteal uptake especially within the tibial shafts (blue arrows).

Plain chest X‐ray indicated long‐standing bibasal changes of bronchiectasis without evidence of acute consolidation. Computerized tomography of the thorax revealed extensive bronchiectasis (Fig. 2) with relative sparing of the upper lobes. There was no evidence of lung malignancy. Plain radiographs showed extensive periosteal reaction involving the appendicular skeleton especially the distal radius and ulna (Fig. 3). Whole‐body bone scan showed generalized tracer uptake in the cortices of the extremities especially the distal tibias (Fig. 4). Skeletal radiological appearances were classical for HPOA.

He was treated for an infective exacerbation of bronchiectasis with a four‐week course of intravenous antibiotics (cefuroxime), and a seven‐day course of prednisone 20 mg daily to help alleviate his joint pain. As his respiratory symptoms and inflammatory markers improved, there was corresponding improvement in his joint symptoms. He reported more rapid improvement in his joint pain with prednisone compared to previous episodes. Over the subsequent two‐month period following discharge, he had two further infective exacerbations of his bronchiectasis requiring hospitalization and again had recurrent joint pain and swelling of the wrists, knees, and ankles. Similarly, this joint pain resolved together with his respiratory symptoms following antibiotic therapy.

## Discussion

The syndrome of HPOA is associated with a wide spectrum of conditions and are generally separated into malignant and non‐malignant aetiologies [3]. The most common non‐malignant disease is CF, where it is reported to occur in 2–7% of patients [2, 4].

HPOA associated with bronchiectasis has not previously been described in an adult and in our patient, this resulted in recurrent joint pain with serial infective exacerbations of bronchiectasis. Despite the relationship to CF, HPOA related to bronchiectasis has only been reported in one other case, in an adolescent, who experienced recurrent episodes of arthralgia during pulmonary infections. Similar to our case, effective treatment of the underlying pulmonary infection with intravenous antibiotics resulted in improvement of the joint symptoms [5]. This association of HPOA with pulmonary exacerbations in CF is well recognized; aggressive treatment of exacerbations with intravenous antibiotics and optimization of pulmonary status usually improves the symptoms of HPOA [2].

The exact pathogenesis of HPOA is unknown but different hypotheses have been proposed; the two main models suggest neurogenic and humoral mechanisms. The neurogenic model is based on observations that symptoms of HPOA can be relieved by chemical or surgical vagotomy [1]. The humoral model focuses on accumulation of growth factors in the peripheral circulation, due to pathological intrapulmonary shunting with resultant failure of fragmentation of platelets and megakaryocytes; these then clump and are deposited in the distal vasculature, where they release vasoactive compounds such as vascular endothelial growth factor (VEGF), platelet‐derived growth factor (PDGF), and prostaglandin E_2_. In turn, this promotes angiogenesis, fibroblast proliferation, and new soft tissue and bone formation [1, 3]. These mechanisms may be triggered by the presence of chronic inflammation, persistent infection, hypoxia, and malignancy [6].

Although HPOA is associated with CF, it is peculiar that it is not recognized as a complication of non‐CF bronchiectasis. Our patient had chronic airway infection with *P. mirabilis*, which is not commonly isolated from the sputum of patients with bronchiectasis. His symptoms responded to treatment, primarily with antibiotics, during each exacerbation. We speculate that this organism may have contributed to the development of HPOA, although the specific mechanism for this is unclear. Of interest, *P. mirabilis* infection is known to induce cross‐reactive autoantibodies that are associated with the development of arthritis [7].

Treatment for HPOA can be classified into treatment of the primary cause (e.g. tumour resection) and symptomatic management. Symptomatic treatment include non‐steroidal anti‐inflammatory drugs (NSAIDs), bisphosphonates, octreotide, and vagotomy, each with varying levels of evidence [3]. Our patient was treated with a short course of oral glucocorticoids (prednisone) as an anti‐inflammatory agent for his pain. His joint symptoms improved and prednisone may have accelerated this response.

In summary, we report a rare case of recurrent HPOA in an adult patient with bronchiectasis, who experienced deterioration in joint symptoms during each acute exacerbation of bronchiectasis. HPOA should be considered in patients with bronchiectasis and coexistent arthralgia.

### Disclosure Statement

Appropriate written informed consent was obtained for publication of this case report and accompanying images.
